# Cognitive Difficulties and Health-Related Quality of Life in Sarcoidosis: An Analysis of the GRADS Cohort

**DOI:** 10.3390/jcm11133594

**Published:** 2022-06-22

**Authors:** Karin F. Hoth, Jacob Simmering, Anna Croghan, Nabeel Y. Hamzeh

**Affiliations:** 1 Department of Psychiatry, University of Iowa, Iowa City, IA 52242, USA; karin-hoth@uiowa.edu (K.F.H.); anna-croghan@uiowa.edu (A.C.); 2 Iowa Neuroscience Institute, University of Iowa, Iowa City, IA 52242, USA; 3 Department of Internal Medicine, University of Iowa, Iowa City, IA 52242, USA; jacob-simmering@uiowa.edu

**Keywords:** sarcoidosis, fatigue, quality of life, cognition, cognitive difficulties, health-related quality of life

## Abstract

Rationale: Subjective cognitive difficulties are common among sarcoidosis patients; however, previous studies have not modeled the link between cognitive difficulties and health-related quality of life (HRQOL). Objectives: To determine whether cognitive difficulties are associated with HRQOL in sarcoidosis patients after adjusting for demographics, fatigue, and physical disease severity measures. Methods: We performed a secondary analysis of the Genomic Research in Alpha-1 antitrypsin Deficiency and Sarcoidosis (GRADS) study data. We examined the association between self-reported cognitive difficulties (Cognitive Failures Questionnaire (CFQ)) and HRQOL (SF12v2 mental and physical component scores) while adjusting for the demographics, fatigue, and physical disease severity measures (i.e., organ involvement, forced vital capacity). Results: Approximately one-fourth of the patients with sarcoidosis endorsed cognitive difficulties. More frequent cognitive difficulties and more severe fatigue were significantly associated with worse mental HRQOL in the fully adjusted model, while older age was associated with better mental HRQOL. The association between cognitive difficulties and physical HRQOL was not significant in the final model. More severe fatigue, joint involvement, and reduced forced vital capacity (FVC) were associated with worse physical HRQOL, while higher income and higher education were associated with better physical HRQOL. Conclusions: Perceived cognitive difficulties are associated with diminished HRQOL after adjusting for demographics, organ involvement, pulmonary function, and fatigue. The association between cognitive difficulties and reduced HRQOL primarily occurs through the impact on mental components of HRQOL.

## 1. Introduction

Sarcoidosis is a multi-systemic inflammatory disease that predominantly involves the lungs and lymph nodes, although any organ can be affected [1]. Symptoms related to sarcoidosis differ across patients and organ involvement but often include cough, fatigue, and shortness of breath, but can also be non-organ specific such as weight loss, pain, and cognitive impairment [1]. Individuals with sarcoidosis are also at increased risk of depression and cognitive complaints [2,3]. The course of disease is variable, with some patients experiencing progression and others resolution of symptoms, adding uncertainty to the impact of the disease on individuals’ lives [1]. Sarcoidosis typically begins in early to middle adulthood, striking at a time of peak occupational productivity and family responsibilities [4].

Previous research has shown that patients with sarcoidosis have a reduced quality of life (QOL) compared to age-matched peers [5,6,7,8,9,10,11,12]. Several factors that may impact QOL have been examined, including patients’ demographic characteristics (e.g., female gender [13,14,15], lower income [16]), severity of depressive symptoms and anxiety [9], fatigue [17,18], pain, specific organ involvement [19], and medication effects [20,21]). This research has been chiefly conducted at single centers. Overall, relatively few studies in sarcoidosis have focused on patient-centered clinical outcomes, leading to several recent calls in the literature for more research into the causes of disease burden [22,23]. Identifying potentially modifiable co-morbidities that impact health-related quality of life has not previously been an area of focus but would open new avenues for interventions to improve daily life for patients.

Cognitive difficulties are one set of symptoms that are likely to impact QOL but have not been well-studied in sarcoidosis [22,23,24,25]. Initial research suggests that approximately one-third of patients with sarcoidosis report cognitive problems in their everyday life [24], as measured using the Cognitive Failures Questionnaire (CFQ) [26]. Elfferich and colleagues found that patients with sarcoidosis reported increased cognitive symptoms over the previous five years, greater worry about cognitive symptoms, and greater hindrance from cognitive difficulties in daily life than their peers [24]. Research from the same group observed that high CFQ scores, which reflect patients’ experience of more frequent cognitive errors, were associated with female gender [24], younger age [25], fatigue [24,25], depressive symptoms, and symptoms of small-fiber neuropathy [24], many of the same factors that have been related to poor QOL in the past literature. To date, no study has modeled the potential link between cognitive difficulties and health-related quality of life while considering demographics, fatigue, and indicators of sarcoidosis severity. As such, further research is needed to better understand the extent and complexity of factors impacting health-related quality of life of patients with sarcoidosis [22,23].

The primary goal of the current study was to determine whether cognitive difficulties are associated with reduced HRQOL in patients with sarcoidosis above and beyond that explained by key demographic and clinical characteristics, such as fatigue. We examined the association between cognitive difficulties and HRQOL among the 315 patients with complete data on relevant measures at the GRADS baseline visit. We hypothesized that more frequent cognitive difficulties would be associated with poorer HRQOL. Further, we hypothesized that cognitive difficulties would have a unique, independent association with HRQOL after adjusting for demographic characteristics, fatigue, and severity of sarcoidosis, measured by lung function and type of organ involvement. Finally, we hypothesized that more frequent cognitive difficulties would have a stronger association with the Mental Component of HRQOL than the Physical Component, as measured by the SF-12.

## 2. Methods

The current study is a secondary analysis of the questionnaires and clinical data collected at the baseline visit of the Genomic Research in Alpha-1 antitrypsin Deficiency and Sarcoidosis (GRADS) study [27]. GRADS was an NIH-sponsored, multi-center, observational cohort study designed to investigate the lung microbiome and genomics of alpha-1 anti-trypsin deficiency and sarcoidosis [27]. Our study was granted a waiver of consent by the University of Iowa institutional review board, as it was a secondary analysis of de-identified data.

Inclusion criteria for the parent GRADS study required that participants with sarcoidosis be at least 18 years old, diagnosed with sarcoidosis based on the American Thoracic Society (ATS), European Respiratory Society (ERS)/World Association of Sarcoidosis and Other Granulomatous diseases (WASOG) criteria [28], able and willing to undergo GRADS study procedures, and capable of understanding study forms and providing consent [27]. Eligible participants completed self-administered questionnaires regarding their demographics and medical history, fatigue (PROMIS), cognitive difficulties (CFQ), and HRQOL (Medical Outcomes Study 12-Item Short-Form Health Survey, SF-12v2) [27]. Participants also completed pulmonary function testing including spirometry and diffusion capacity and chest imaging, and each site investigator completed medical questionnaires based on a review of each participants’ medical record to document key aspects of sarcoidosis disease phenotype along with current and past medical treatment. Full details of the GRADS study design have been previously published [27].

## 3. Measures

Demographic and medical history questionnaires: Participants self-reported their race, age, gender, level of education, income, medical history, and medication use, including current immunosuppressive regimens.

Sarcoidosis disease status and history: Study site investigators completed an organ assessment form (GRADS organ assessment) which was developed by the sarcoidosis protocol committee based on previously published organ assessment tools [29,30].

Spirometry: Pre- and post-bronchodilator spirometry and diffusion capacity were obtained at baseline according to the ATS/ERS guidelines [31].

Quality of life: The SF-12v2 was administered as a measure of HRQOL. It is a self-report questionnaire that reflects functional health and well-being. The SF-12 was developed as a shorter alternative to the longer SF-36. The SF-12v2 physical component summary score (PCS) and mental component summary score (MCS) were calculated by the genomic information center for the GRADS study according to standard guidelines. The PSC and MCS range from 0 to 100, with higher scores indicating better HRQOL [32]. The SF-36 has been previously utilized to assess HRQOL in sarcoidosis patients [11]. The Fatigue Assessment Scale (FAS) was also collected in GRADS but a large number of the FAS questionnaires had missing answers, impairing our ability to calculate a score.

Cognitive difficulties: Perceived cognitive difficulties were measured using the Cognitive Failures Questionnaire (CFQ), a self-report questionnaire measure that has been utilized in previous sarcoidosis research [24,25]. The CFQ was developed in 1982 [26] and includes 25 items that describe everyday cognitive errors (e.g., forgetting names, failure to notice signposts, confusing left and right). Individuals rate the frequency with which they experience cognitive errors on a 5-point Likert scale from “never” to “very often” [26]. The total scores range between 0 and 100, with higher scores reflecting a report of more frequent cognitive errors in everyday life [26]. The CFQ has been further tested and validated in other cohorts [33,34,35]. To describe our sample, we determined the number of patients who scored above the previously published cutoff for “elevated cognitive deficits” in the past literature of 43 or greater [24]. However, in our primary statistical models, we included the CFQ score as a continuous measure to capture the full variability across the questionnaire.

Fatigue: Ten items from the Patient-Reported Outcomes Measurement Information System (PROMIS) fatigue databank were used to assess fatigue in the GRADS parent study. PROMIS fatigue items require the participant to rate the frequency of symptoms of fatigue over the past 7 days on a 5-point Likert scale ranging from “never” to “always.” Total scores on the PROMIS fatigue measure have a possible range of 10–50, with higher score indicating more fatigue. The 10 items used in the GRADS study are presented in Supplementary Table S1. There are no established clinical cutoffs for the 10-item fatigue questionnaire in patients with sarcoidosis, thus we opted to include the total score as a continuous variable in our analysis.

## 4. Data Analysis

Descriptive statistics including frequency distribution, median, and interquartile range were used to describe the demographic and clinical characteristics of the sample. Our primary analyses utilized ordinary least squares regression modeling to examine the association between cognitive difficulties (as measured by the CFQ) and HRQOL, while adjusting for key demographic and clinical variables selected a priori based on the past literature demonstrating an association with HRQOL. These independent variables included: demographic characteristics (age, gender, race), socio-economic status (education, self-reported income split into four roughly equal groups), fatigue (measured using PROMIS Fatigue total score), and sarcoidosis disease factors. We included dummy variables for organ system involvement for any system with at least 10% prevalence (lung, bone, eye, cardiac, joint, lymphatic), the sum of the other organ systems with less than 10% prevalence, and the FVC percent predicted to describe sarcoidosis severity. We conducted three regression models in total, with HRQOL serving as the dependent variable (SF-12 total score, SF-12 mental component score (MCS) alone, and SF-12 physical (PCS) component score, respectively). Analysis was performed utilizing R foundation for statistical computing (Vienna, Austria).

## 5. Results

### 5.1. Patient Characteristics

**Figure 1:**
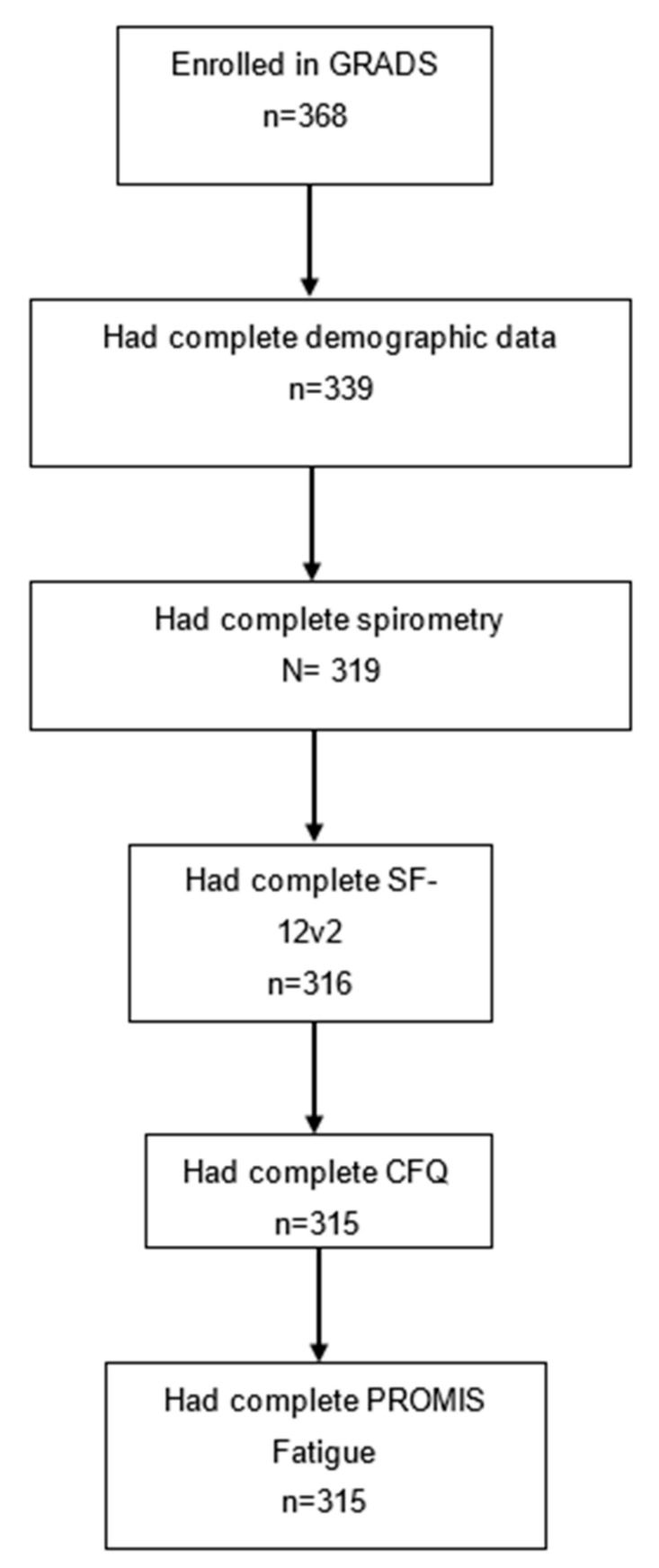
Consort diagram of GRADS participants included in the current analysis based on available data.

GRADS recruited 368 patients with sarcoidosis from 9 sarcoidosis centers across the United States [27]. For the current analysis, we excluded participants with incomplete data for the key variables (i.e., demographics, spirometry, SF-12v2, and CFQ), and a consort diagram depicting the sample size is shown in Figure 1. Our final sample consisted of 315 individuals with an average age of 53 years. Slightly over half of the participants were female (54%), with the majority identifying as white (72%) and reporting at least some college education (81%). Demographic and clinical characteristics are detailed in Table 1.

**Figure 2:**
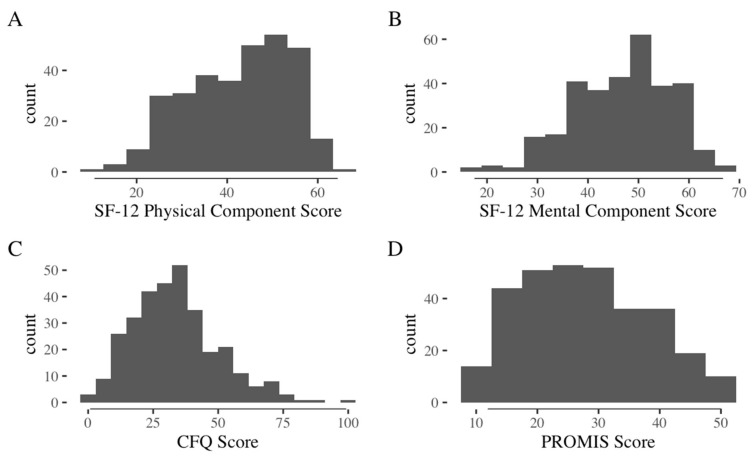
Distribution of SF-12v2, CFQ, and PROMIS Fatigue scores for the entire sample (*n* = 315).

Means, standard deviations (SD), and score distributions for key independent variables (CFQ and PROMIS Fatigue) and HRQOL outcomes (SF-12v2) are presented in Table 1 and Figure 2, respectively. The overall mean ± SD CFQ score for the sample was 34 ± 17, with a median of 33 and interquartile range of 22 to 42. A CFQ score of 43 or more has been defined in past literature as clinically significant perceived cognitive difficulties [24], and 25% of our cohort scored 43 or above. The mean ± SD for the MCS of the SF-12v2 was 47 ± 10, whereas the PCS was 42 ± 11. The total SF-12v2 score (MCS + PCS) was 89 ± 16.

### 5.2. Association between Cognitive Difficulties and Quality of Life

Overall HRQOL was described using a regression model with SF-12v2 total score as the dependent variable. The overall model was significant (F = 30.4, *p* < 0.001; see Table 2). There was a statistically significant relationship between CFQ (β = −0.08, *p* = 0.041) and fatigue (β = −1.02, *p* < 0.001) and lower overall HRQOL that persisted after adjustment for age, race, education, income, disease severity, and FVC.

The ordinary least squares regression model with mental HRQOL (SF-12v2 MCS) as the dependent variable was significant overall (F = 9.73, *p* < 0.001; see Table 2). There was a significant association between more frequent cognitive difficulties (β = −0.12, *p* < 0.001) and fatigue (β = −0.39, *p* < 0.001) and SF-12v2 MCS after adjusting for all other covariates. Older age was also associated with better mental HRQOL (β = 1.43, *p* = 0.002).

The regression model with physical HRQOL (SF-12v2 PCS) as the dependent variable was also significant overall (F= 13.9, *p* < 0.001; see Table 2); however, in contrast with mental QOL, cognitive difficulties was not significantly associated with physical HRQOL in the adjusted model (β = 0.04, *p* = 0.311). Fatigue remained statistically significantly associated with SF-12v2 PCS (β = −0.63, *p* < 0.001). Higher income and education levels were associated with higher SF-12v2 PCS scores, while joint involvement and reduced FVC were associated with worse physical HRQOL.

Additional specifications explicitly including medication use, medication type, disease duration, flags for SFN, or neurological involvement found no meaningful changes in the relationship between CFQ and SF-12 overall or in either sub-score.

## 6. Discussion

The current study highlights the extent and importance of self-reported cognitive difficulties in patients with sarcoidosis. Consistent with prior estimates [24], approximately one quarter of our cohort of 315 patients with sarcoidosis scored above the previously published cutoff for subjective cognitive dysfunction on a commonly used cognitive questionnaire measure (i.e., CFQ of 43 or above) [24]. The primary new finding of the study is that perceived cognitive difficulties are associated with worse HRQOL after adjusting for the effects of demographic characteristics, organ involvement, pulmonary function, and severity of fatigue, which have previously been examined in relation to quality of life in sarcoidosis. The association between cognitive difficulties and worse HRQOL appeared to primarily occur through the impact on mental components of HRQOL (i.e., SF-12v2 MCS), while fatigue was associated with both mental and physical HRQOL items.

Fatigue and subjective cognitive difficulties often co-occur. Prior research has demonstrated an association between both mental and physical fatigue and self-reported cognitive failures in neurosarcoidosis patients [36]. However, fatigue and cognitive impairment are distinct symptom clusters. Fatigue, along with exercise capacity, have also been found to relate to physical QOL in sarcoidosis patients [37]. Notably, the current findings suggest that patients’ perceived cognitive difficulties are independently associated with HRQOL beyond fatigue and other clinical characteristics. Thus, clinicians should ask patients about cognitive symptoms and consider their potential impact on daily functioning. Future research may benefit from including both measures of fatigue and cognitive difficulties to optimally understand patient-reported and clinical outcomes. One previous treatment trial of 343 patients that included questions regarding both fatigue and cognitive concerns showed that patients with sarcoidosis who had a high frequency of cognitive difficulties (i.e., CFQ ≥ 43) also had high fatigue scores, and that both fatigue and perceived cognitive difficulties improved in patients treated with an anti-TNF-α agent [24]. This underscores the potential utility of adding cognitive difficulties as an additional endpoint in clinical trials.

In addition to findings for cognitive difficulties and fatigue, our analyses identified several demographic and clinical characteristics that are associated with HRQOL in our sample. Older age was associated with better mental HRQOL, an observation that is consistent with findings in other chronic pulmonary conditions [38]. Unsurprisingly, poorer pulmonary function measured by FVC percent predicted was associated with worse physical HRQOL. Lower income was also associated with worse physical HRQOL, while education above a bachelor’s degree appeared protective and was related to better physical HRQOL. Lower income associations with reduced HRQOL have been found in other studies [16]. For example, one study analyzing the Sarcoidosis Advanced Registry for Cures database, which was established by the Foundation for Sarcoidosis Research, showed that patients with low income had higher rates of sarcoidosis-related co-morbidities and lower HRQOL based on the Sarcoidosis Health Questionnaire [16].

## 7. Limitations of the Study

It is important to consider our findings in the context of the study’s strengths and limitations. A key strength is that we included a large and diverse sample of patients with sarcoidosis from the GRADS study. GRADS was a multi-center study involving nine centers across the US that recruited a geographically and clinically diverse sample of patients with sarcoidosis with the goal of representing sarcoidosis in the general population. GRADS did not include a control group for comparison, limiting our ability to compare our findings to a control group at this time. Additionally, our approach to considering fatigue and cognitive difficulties together in our model allowed us to examine whether cognitive difficulties accounted for independent variance in HRQOL. Limitations include the fact that the current analysis is retrospective in nature. Additionally, the GRADS parent study was not focused on assessing QOL measures, as potentially relevant data on depression [39,40], anxiety [40], pain [41], and sleep disorders [42] were not collected. In some instances, such as small-fiber neuropathy, data were collected; however, the GRADS criterion for having small-fiber neuropathy was very high and likely to be met through routine clinical practice. Although GRADS was inclusive of a broad phenotype of sarcoidosis, the recruitment and data collection choices limit the generalizability of our findings to the overall sarcoidosis population [27]. Further, using a self-report questionnaire to assess cognitive difficulties captures patients’ perception of function in everyday life but does not objectively assess cognitive skills as with neuropsychological testing. Finally, while the SF-12 is a well-established measure of HRQL, it assesses self-rated mental and physical health and the degree to which health status impacts engagement in various activities rather than life satisfaction. Inclusion of a life satisfaction measure in future studies would be helpful in gaining a more comprehensive appreciation of how QOL is affected in sarcoidosis patients.

Cognitive difficulties in sarcoidosis have primarily been subjectively assessed in past research using self-administered questionnaires and seldom by objective testing [43]. It is well-established in the behavioral neuroscience literature that subjective cognitive complaints are only modestly related to objective cognitive defects due in part to co-morbid conditions such as depression, anxiety, sleep disorders, and medication side effects, which can complicate the overall assessment and management of cognitive impairment in sarcoidosis. Self-report about perceived cognitive complaints does not provide the same information as objective assessment. As reported by Hendriks and colleagues, a pilot study examining subjective and objective cognitive functioning in a limited sample of sarcoidosis patients (*n* = 27) found that self-reported cognitive failures did not significantly relate to performance on objective neuropsychological measures [25]. Perception is based on expectations and, in part, is often associated with other symptoms such as depression. Nonetheless, in some other chronic medical conditions, patient reports of cognitive difficulties have been associated with subsequent development of impairment, and perceived difficulty itself is associated with a loss of function [44]. Longitudinal data including both measures of perceived deficits and objective performance will be needed to determine if this is also true in sarcoidosis. Objective assessment of cognition in future research will be important; however, comprehensive cognitive evaluation is time-consuming and typically cannot be accomplished within the time boundaries of a normal clinic visit. Thus, new efficient assessment tools are also needed to aid physicians in identifying and quantifying cognitive deficits in sarcoidosis and to understand the contribution of co-morbid conditions to these symptoms. Ultimately, clinical management of cognitive difficulties will require a multi-modal, multi-disciplinary approach. Finally, the etiology of cognitive difficulties in sarcoidosis is not well-understood. Further work to identify the underlying biological mechanisms of cognitive impairment is needed.

## 8. Summary

In summary, our study suggests that cognitive difficulties are associated with HRQOL in patients with sarcoidosis after adjusting for important demographic and clinical factors. Perceived cognitive difficulties are associated with diminished HRQOL after adjusting for demographics, organ involvement, pulmonary function, and fatigue. The association between cognitive difficulties and reduced HRQOL primarily occurs through the impact on mental components of HRQOL. Future studies that include measures of perceived difficulties, measures of other relevant factors such as pain and depression, and objective cognitive performance are needed. Examining the relationship between cognitive dysfunction and co-morbid conditions using physiological measures to identify potential mechanisms will move the field toward the study of targeted interventions.

## Figures and Tables

**Table 1:** Demographic and clinical characteristics of the sample (*n* = 315).

	Mean (SD) or *n* (%)
Demographics	
Age (years)	53 (10)
Gender (female)	169 (54%)
Education	
High School or Less	59 (19%)
Some College to Associates Degree	110 (35%)
Bachelor’s Degree or More	146 (46%)
Race	
White	228 (72%)
Black	75 (24%)
Other	12 (3.8%)
Income (in US dollars)	
0 to 49,999	96 (30%)
50,000 to 99,999	91 (29%)
100,000 to 149,999	63 (20%)
150,000 or more	65 (21%)
**Clinical Characteristics**	
Disease duration (years from diagnosis to enrollment) *	5 (2, 12)
Sarcoidosis organ involvement by physician rating	
Lung involvement (Yes)	307 (97%)
Skin involvement (Yes)	83 (26%)
Eye involvement (Yes)	55 (17%)
Cardiac involvement (Yes)	60 (19%)
Joint involvement (Yes)	56 (18%)
Lymph involvement (Yes)	53 (17%)
Liver involvement (Yes)	28 (8%)
Ear, nose, and throat involvement (Yes)	27 (9%)
Glandular involvement (Yes)	27 (9%)
Neurological involvement (Yes)	21 (7%)
Bone involvement (Yes)	13 (4%)
Renal involvement (Yes)	9 (3%)
Small fiber neuropathy (Yes)	11 (4%)
Number of Organ Systems Affected	2.4 (1.5)
Forced Vital Capacity (FVC) % predicted	87 (18)
Diffusion Capacity for Carbon monoxide (DLCO) % predicted	79 (26)
Scadding Stage (0/I/II/III/IV/missing)	38/62/93/45/76/1
Medication treatment	
Current treatment with steroid-sparing agents	72 (23%)
Current treatment with prednisone	84 (27%)
Current treatment with anti-TNF-alpha	14 (5%)
Current treatment with other immunosuppressants	39 (12%)
**Questionnaire Measures**	
Cognitive Failure Questionnaire (CFQ) Total Score	34 (17)
CFQ Score ≥ 43	78 (25%)
PROMIS Fatigue Total Score	28 (10)
SF-12v2	
Total Score	89 (16)
Mental Component Score (MCS)	47 (10)
Physical Component Score (PCS)	42 (11)

SF-12 = Medical Outcomes Study 12-Item Short-Form Health Survey, PROMIS: Patient-Reported Outcomes Measurement Information System. * Median and interquartile ranges were used to describe duration of disease due to a large skew when using mean and standard deviation.

**Table 2:** Associations between demographic and clincial variables and health-related quality of life subscales (SF-12 MCS and PCS): results of linear regression analyses (N = 315).

Predictor Variable	SF-12 Total Score Overall Model: F = 30.4, *p* < 0.001				SF-12 Mental Component Score Overall Model: F = 9.7, *p* < 0.001				SF-12 Physical Component Score Overall Model: F = 13.9, *p* < 0.001			
	Unstand. b	β	SE	*p*	Unstand. b	β	SE	*p*	Unstand. b	β	SE	*p*
Cognitive Difficulties (CFQ Score)	**−0.08**	**−0.09**	**0.04**	**0.041**	**−0.12**	**−0.21**	**0.03**	**<0.001**	0.04	0.05	0.04	0.311
Fatigue (PROMIS Score)	**−1.02**	**−0.67**	**0.06**	**<0.001**	**−0.39**	**−0.42**	**0.05**	**<0.001**	**−0.63**	**−0.57**	**0.06**	**<0.001**
Age (per decade)	0.85	0.05	0.56	0.129	**1.43**	**0.15**	**0.46**	**0.002**	−0.58	−0.05	0.51	0.256
Female Gender	1.22	0.04	1.13	0.281	0.76	0.04	0.94	0.417	0.46	0.02	1.03	0.654
Race												
White	Reference				Reference				Reference			
Black	−2.27	−0.06	1.39	0.105	−0.04	0.00	1.15	0.970	−2.23	−0.08	1.27	0.080
Other	1.73	0.02	2.83	0.542	2.00	0.04	2.34	0.393	−0.28	0.00	2.57	0.915
Income												
0 to 49,999	Reference				Reference				Reference			
50,000 to 99,999	1.49	0.04	1.46	0.308	−1.14	−0.05	1.21	0.346	**2.63**	**0.11**	**1.32**	**0.048**
100,000 to 149,999	**3.93**	**0.10**	**1.68**	**0.020**	1.27	0.05	1.39	0.363	2.67	0.09	1.53	0.082
150,000 or more	3.55	0.09	1.82	0.053	−0.89	−0.04	1.51	0.554	**4.44**	**0.16**	**1.66**	**0.008**
Education												
High School or Less	Reference				Reference				Reference			
Some College to Associates	1.84	0.06	1.56	0.238	1.15	0.06	1.29	0.374	0.69	0.03	1.42	0.625
Bachelor’s Degree or More	**4.23**	**0.14**	**1.62**	**0.009**	1.09	0.06	1.34	0.415	**3.14**	**0.14**	**1.47**	**0.033**
Presence of Lung Involvement	1.79	0.02	3.41	0.600	−0.05	0.00	2.82	0.986	1.84	0.03	3.10	0.553
Presence of Bone Involvement	0.85	0.01	3.05	0.780	−1.21	−0.03	2.52	0.632	2.06	0.04	2.77	0.458
Presence of Eye Involvement	0.42	0.01	1.55	0.788	−0.43	−0.02	1.29	0.736	0.85	0.03	1.41	0.547
Presence of Cardiac Involvement	−0.07	0.00	1.37	0.960	0.74	0.03	1.14	0.516	−0.81	−0.03	1.25	0.518
Presence of Joint Involvement	−2.10	−0.05	1.49	0.161	0.91	0.04	1.24	0.462	**−3.01**	**−0.10**	**1.36**	**0.027**
Presence of Lymph Involvement	−1.76	−0.04	1.51	0.246	−0.70	−0.03	1.25	0.575	−1.06	−0.03	1.38	0.443
Number of Organ Systems Affected	−0.98	−0.04	0.94	0.300	−0.29	−0.02	0.78	0.709	−0.69	−0.04	0.86	0.422
FVC % Predicted (per 10%)	**0.98**	**0.11**	**0.32**	**0.002**	−0.08	−0.02	0.26	0.755	**1.06**	**0.17**	**0.29**	**<0.001**

Bold font indicates *p* < 0.05. FVC = Forced Vital Capacity, CFQ = Cognitive Failures Questionnaire, SF-12 = Medical Outcomes Study 12-Item Short-Form Health Survey, MCS = Mental Component Score.

## Supplementary Materials

The following supporting information can be downloaded at: https://www.mdpi.com/article/10.3390/jcm11133594/s1.

## Abbreviations

HRQOL	Health-related quality of life
CFQ	Cognitive Failures Questionnaire
GRADS	Genomic Research in Alpha-1 anti-trypsin Deficiency and Sarcoidosis
NIH	National Institute of Health
ATS	American Thoracic Society
ERS	European Respiratory Society
WASOG	World Association of Sarcoidosis and Other Granulomatous diseases
SF-12v2	Medical Outcomes Study 12-Item Short-Form Health Survey version 2
PCS	Physical Component Score
MCS	Mental Component Score
PFT	Pulmonary function test
FVC	Forced Vital Capacity
DLCO	Diffusion Capacity for Carbon Monoxide
TNF-α	Tumor necrosis factor alpha
