# Supplementary material for: Cognitive Difficulties and Health-Related Quality of Life in Sarcoidosis: An Analysis of the GRADS Cohort
Source: J Clin Med. 2022 Jun 22;11(13):3594. doi: 10.3390/jcm11133594 (PMC9267453; doi:10.3390/jcm11133594)
Supplement: Supplementary file 1 [file jcm-11-03594-s001.zip › jcm-1702352-supplementary.pdf]

Form Date: __ __ / __ __ / __ __ __ __ GRADS Study ID: __ __ - __ - __ __ __

Entered: __ __/__ __/__ __ __ __ Initials: _____ Visit: ☐Enrollment / Clinic Visit ☐6month

For office use only.

GRADS: PROMISE Fatigue Profile (PF)

Staff ID: __ __ - __ - __ __ __

Please indicate method of data entry:
- ☐ Patient direct entry
- ☐ Coordinator direct entry during interview
- ☐ Coordinator entry from patient-completed hard copy data collection form

DIRECTIONS

(**Please circle or mark one number per line to indicate your response as it applies to the past 7 days.**)

CONCERNS	Not at all	A little bit	Some-what	Quite a bit	Very much
1. I feel fatigued	1	2	3	4	5

(**Please circle or mark one number per line to indicate your response as it applies to the past 7 days.**)

	Never	Rarely	Sometimes	Often	Always
2. How often did you have to push yourself to get things done because you were fatigued?	1	2	3	4	5

(**Please circle or mark one number per line to indicate your response as it applies to the past 7 days.**)

	Not at all	A little bit	Some-what	Quite a bit	Very much
3. How much were you bothered by your fatigue on average?	1	2	3	4	5
4. How run down did you feel on average?	1	2	3	4	5
5. I have trouble starting things because I am tired	1	2	3	4	5
6. To what degree did your fatigue interfere with your physical functioning?	1	2	3	4	5
7. To what degree did your fatigue interfere with your social activities?	1	2	3	4	5
8. I feel listless (“washed out”)	1	2	3	4	5

(**Please circle or mark one number per line to indicate your response as it applies to the past 7 days.**)

	Never	Rarely	Sometimes	Often	Always
9. How often did your fatigue make it difficult to make decisions?	1	2	3	4	5

(**Please circle or mark one number per line to indicate your response as it applies to the past 7 days.**)

	Not at all	A little bit	Some-what	Quite a bit	Very much
10. I have to limit my social activities because I am tired	1	2	3	4	5
